# How frequent are vancomycin-resistant enterococci in patients with primary sclerosing cholangitis and ulcerative colitis treated with oral vancomycin?

**DOI:** 10.1007/s12664-022-01286-9

**Published:** 2022-10-10

**Authors:** Ayesha Shah, Sahar Pakneeshan, Michael P Jones, Natasha Koloski, Gavin Callaghan, Mark Morrison, Gerald Holtmann

**Affiliations:** 1 Faculty of Medicine and Faculty of Health and Behavioral Sciences, The University of Queensland, Brisbane, QLD Australia; 2 Department of Gastroenterology and Hepatology, Princess Alexandra Hospital, Ipswich Road, Woolloongabba, Brisbane, QLD Australia; 3 AGIRA (Australian Gastrointestinal Research Alliance) and the NHMRC Centre of Research Excellence in Digestive Health, Brisbane, Australia; 4 Department of Psychology, Macquarie University, Sydney, NSW Australia; 5 University of Queensland Diamantina Institute, Woolloongabba, QLD Australia

**Keywords:** Antibiotics, Autoimmune liver disease, Colitis, Crohn’s disease, Inflammatory bowel disease, Oral vancomycin therapy, Primary sclerosing cholangitis, Ulcerative colitis, Vancomycin-resistant enterococci

## Abstract

In patients with primary sclerosing cholangitis (PSC), antimicrobial therapy with oral vancomycin (OV) is increasingly used to prevent progression of the liver disease and control concomitant ulcerative colitis (UC); however, there are concerns regarding the risk of development of vancomycin-resistant enterococci (VRE). Thus, we aimed to determine the incidence of VRE in PSC-UC patients. We conducted a retrospective study of PSC-UC patients, treated with OV at the Department of Gastroenterology at the Princess Alexandra Hospital. VRE testing was performed utilizing rectal swabs. We included 7 PSC-UC patients (age 22–53 years, 2 females) treated with OV with daily dose ranging from 250 to 1500 mg. All patients were treated for at least 6 months with OV (range 9–31 months, mean 32.1 months). All patients achieved complete clinical remission of the UC, with mean reduction of fecal calprotectin by 634 μg/mg (87.3%), mean reduction in the C-reactive protein by 21.9 mg/L (74.2%), and mean reduction in the total Mayo score by 9.3 (93.3%). With regard to the liver parameters, mean improvement in alkaline phosphatase enzyme and total bilirubin was −48.7 U/L (−19.7%) and −2.7 mg/dL (−19.6%), respectively. No patient treated with OV developed VRE or reported any adverse events. This cohort study including PSC-UC patients did not provide evidence for development of VRE, while treatment with vancomycin was associated with clinical and endoscopic remission of the UC. Larger, prospective trials are required to define the efficacy and safety of antimicrobial therapy in PSC-UC, while the risk of VRE appears small.

Bullet points of the study highlights ***What is already known?***

- Oral vancomycin (OV) prevents progression in primary sclerosing cholangitis (PSC) and controls concomitant ulcerative colitis (UC).
- However, data on vancomycin-resistant enterococcus (VRE) are lacking.

***What is new in this study?***

- In patients with PSC-UC, with long-term treatment with OV, sustained complete UC remission was achieved.
- Also, during long-term follow-up, no VRE was observed.

***What are the future clinical and research implications of the study findings?***

- OV appears to be safe and effective for the treatment of PSC-UC. No VRE was observed in the treatment cohort. These results need to be confirmed in larger prospective, placebo controlled randomized controlled trials.

## Introduction

Primary sclerosing cholangitis (PSC) is a rare, chronic cholestatic disease that is characterized by inflammation and fibrosis of the large and small bile ducts, which can lead to fibrosis involving the hepatic parenchyma and biliary tree, cirrhosis, and end-stage liver disease [1]. PSC is an immune-mediated disorder that is often associated with inflammatory bowel disease (IBD), with 60% to 70% of patients with PSC having coexisting IBD, most commonly ulcerative colitis (UC). PSC is associated with IBD in 70% to 80% of patients in the west as compared to 20% to 30% in Asia [2]. Notably, UC associated with PSC is unique and differs from the typical phenotype of UC, manifesting as pancolitis, with a right-sided predominance, rectal sparing, and backwash ileitis [3]. Although PSC was first described in medical literature in 1857, the etiology of PSC remains incompletely understood, which in part explains a lack of effective or proven medical therapy for this condition. Despite being an immune-mediated disease, treatment with immune suppressants does not appear to alter the course of disease or cure PSC, and liver transplantation is so far the only proven therapy to extend life expectancy [1].

Although there is no curative treatment for PSC, there is emerging evidence that targeted modulation of the gastrointestinal microbiome by antibiotic therapy appears to alter the natural course of PSC and delay the progression of disease. In our recently published systematic review and meta-analysis [4], we found in adult PSC patients with or without an associated IBD, short-term treatment with antimicrobial therapy, in particular with oral vancomycin (OV), was associated with significant improvement in cholestatic liver enzymes and PSC Mayo risk score (MRS). More recently, Dao et al. [5] found that OV was effective for the induction and maintenance of remission of UC in adults with UC-PSC, including PSC patients having undergone orthoptic liver transplantation (OLT). We describe here our experience in a cohort of well-characterized adult PSC patients (both pre- and post-OLT), with an associated IBD, who were treated with OV therapy for management of colitis, specifically having failed to respond to conventional medical therapy for management of IBD. Additionally, potential adverse effects, including the development of vancomycin-resistant enterococcus (VRE), were carefully monitored.

##  Methods

We conducted a retrospective audit of well-characterized cohort of consecutive adult patients with an established diagnosis of PSC and concomitant IBD, who were treated with OV at the Department of Gastroenterology and Hepatology at the Princess Alexandra Hospital between July 2015 and May 2021. Oral vancomycin was initiated in the IBD clinic, predominantly for pre-transplant patients with PSC and associated IBD who had failed to respond to conventional medical therapy for management of IBD and for post-transplant patients with PSC and associated IBD as a treatment option for managing colitis, prior to increasing immunosuppression. VRE testing was performed utilizing rectal swabs, employing standard clinical culture-based screening methods [6]. The aim of this study was not to focus on the liver-specific end points. The local ethics committee approved this study (HREC/2021/QMS/76269).

## Results

The study included seven patients with PSC (four post-orthoptic liver transplantation and three pre-transplant) with concomitant UC (six with pancolitis and one with J-pouch). Their age ranged from 22 to 53 years, and out of the seven patients, two were females. All patients were treated for at least 6 months with OV (range 9 to 31 months, mean 32.1 months or cumulated vancomycin exposure 225 months).

**Fig. 1:**
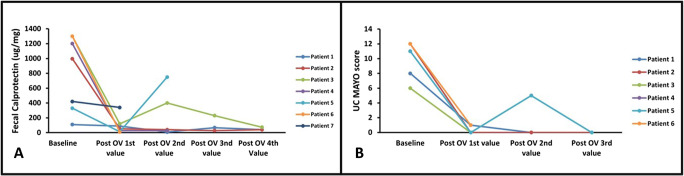
Panel **A** indicates fecal calprotectin at baseline and at various intervals post-oral vancomycin therapy. Patient No. 5 had concomitant viral infection with Rota virus, identified in stool samples using quantitative real-time polymerase chain reaction, was managed conservatively with no further escalation in immunosuppression; no follow-up fecal calprotectin results are available for this patient. Panel **B** indicates ulcerative colitis Mayo score at baseline and at various intervals post-initiation of oral vancomycin therapy.

All patients treated with OV achieved complete clinical remission of the concomitant UC, with mean reduction of fecal calprotectin by 634 μg/mg (87.3%) (Fig. 1, panel A), mean reduction in the C-reactive protein by 21.9 mg/L (74.2%), and mean reduction in the Mayo UC score by 9.3 (93.3%) (Fig. 1, panel B). With regard to the liver parameters, mean improvements of alkaline phosphatase and total bilirubin were 19.7% (48.7 U/L) and 19.6% (2.7 mg/dL), respectively. No patient developed VRE nor reported adverse events during treatment with OV. Six out of seven patients had pre- and post-OV treatment colonoscopies, and in three out of six patients, colonoscopies revealed endoscopic remission on OV with a Mayo endoscopic score of 0 and resolution of histological inflammation (Table 1). Furthermore, all patients treated with OV had clinical remission. No patient developed VRE or experienced any adverse effects related to this treatment.

**Table 1:** Characteristics of patients with primary sclerosing cholangitis and associated inflammatory bowel disease treated with oral vancomycin

No	Patient	UC extent	Failed prior treatment	Current daily dose of OV	Other concomitant treatment	UC Mayo score, pre-OV	UC Mayo score, post-OV	Reduction in UC Mayo score	Baseline FC (μg/mg)	Post-OV FC (μg/mg)	Duration of remission	PSC phenotype/OLT	Baseline ALP (U/L)	Post-OV ALP (U/L)
1	Male 48 y	UC pancolitis	Sulfasalazine, 5-ASA, thiopurines, and vedolizumab	500 mg	Low-dose prednisolone and UDCA	8 (2/2/2/2)	1 (0/0/1/0)	−7	110	15	September 2018 till date	Cirrhotic (CP-A), PHT	815	700
2	Female 22 y	UC pancolitis	5-ASA, thiopurines, and MTX	1.5 gm	None	12 (3/3/3/3)	0 (0/0/0/0)	−12	997	40	May 2014 till date	Non-cirrhotic	202	99
3	Male 47 y	UC pancolitis	Thiopurines	500 mg	Mesalazine, tacrolimus, low-dose prednisolone and UDCA	6 (1/1/2/2)	NA(0/0/NA/0)	−4	1300	120	November 2019–May 2020	OLTx*2, 2011 and 2017	130	88
4	Male 49 y	UC pancolitis	Cyclosporin	1gm	Low-dose prednisolone, low-dose cyclosporin, thiopurine, and mesalazine	11 (3/2/3/3)	0 (0/0/0/0)	−11	1200	26	October 2019 till date	OLTx 2012	92	65
5	Male 53 y	UC pancolitis	Thiopurine	500 mg	Mesalazine and low-dose prednisolone	11 (3/2/3/3)	0 (0/0/0/0)	−11	330	12	July 2015 till date	OLTx*3, 1990, 1998, 2013	138	107
6	Male 43 y	UC pancolitis	5-ASA	250 mg	Nil	12 (3/3/3/3)	1 (0/0/1/0)	−11	1300	NA	October 2020 till date	Non-cirrhotic	172	162
7	Female 38 y	Subtotal colectomy with J pouch 2017	Metronidazole and 5-ASA suppositories, VSL probiotic, antibiotics — metronidazole, ciprofloxacin, infliximab	500 mg	Vedolizumab and tacrolimus	Pre-pouch ileitis and cuffitis	Normal pouch, cuff, mild ileitis	NA	420	340	August 2020 till date	OLT*1, 2014	176	163

*UC* ulcerative colitis, *y* years, *MTX* methotrexate, *UDCA* ursodeoxycholic acid, *ASA* amino salicylates, *OLT* orthoptic liver transplantation, *ALP* alkaline phosphatase, *OV* oral vancomycin, *PHT* portal hypertension, *NA* not applicable, *FC* fecal calprotectin, UC Mayo score expressed as (stool frequency/rectal bleeding/endoscopic findings/physician’s global assessment). All post OLTx patients were on low-dose prednisolone (≤10 mg/day). Also, patient No 1 was on a weaning dose of prednisolone when oral vancomycin therapy was introduced and only one patient (patient No 7) was on a concomitant biologic (vedolizumab)

## Discussion

There is cumulating evidence that in patients with PSC, targeted modulation of the gastrointestinal microbiota with antimicrobial therapy alters the course of disease [4] and delays or even stops progression. In 1959 Rankin et al. [7] for the first time successfully used tetracycline (500 mg once daily for 10 months) in five patients with PSC (then characterized as chronic, progressive pericholangitis) and associated UC caused by portal bacteremia with biochemical and clinical improvement. Since then, several antibiotics including azithromycin, vancomycin, metronidazole, minocycline, tetracycline, and rifaximin have been used as potential therapeutic option for PSC [4]. Among these, OV is a promising treatment, with benefits on the both the liver and the colon in patients with PSC. Cox et al. [8] were the first to use OV in three children with PSC and associated IBD, with improvement in their liver enzymes and symptoms, which recurred with discontinuation of vancomycin. Since then, several studies have shown OV to be effective for treating both PSC and associated IBD [4].

Vancomycin is a glycopeptide antibiotic, which is poorly absorbed after oral administration with bactericidal effect against Gram-positive bacteria and inactive against Gram-negative bacilli, mycobacteria, or fungi [9]. Thus, OV has minimal systemic absorption and concentrates in the intestine. In PSC and associated IBD, the potential mechanism of actions includes [3] (1) selective effect on Gram-positive species due to its relatively narrow antibiotic spectrum; (2) reduction of the hydrophobic secondary bile acids, which are potentially responsible for the right-sided colitis and after absorption through the enterohepatic circulation for mediating the bile duct injury; and (3) immunomodulatory effect of vancomycin via the tumor necrosis factor (TNF)–alpha inflammatory pathways and/or downstream Treg induction.

However, development of VRE remains a major concern and can have a major impact on public health. Based upon the available literature, multiple genes are necessary to generate vancomycin resistance in enterococci and acquisition of VRE colonization does not occur through mutations in susceptible enterococci in the intestinal tract [10]. Thus, treatment with OV does not directly cause VRE, but selective pressure exerted by OV may facilitate an increase in the concentration of exogenously acquired VRE. Indeed, available studies report that de novo vancomycin resistance is very rare in patients treated with vancomycin [10]. None of our patients developed VRE and similar findings were reported in a pediatric cohort study from the Queensland Children’s Hospital, Australia. In this cohort study of 17 pediatric PSC patients with UC who received vancomycin on average for >8 months, none of the patients developed VRE [11].

The results of our cohort study confirm that in patients with PSC and concomitant IBD, treatment with OV is associated with induction and maintenance of clinical remission in all UC patients who had previously failed to respond to conventional therapies of IBD. More than half of the patients treated with OV attained endoscopic and histologic remission of the associated UC. Given none of the patients developed VRE nor experienced any adverse effects with this treatment, OV might be considered in immunosuppressed patients (e.g. after OLT) since this treatment does not add to the burden of systemic immunosuppression in post-OLT PSC patients.

However, some of the limitations of this study need to be acknowledged. This is a retrospective study, with PSC patients both pre- and post-liver transplant with a relatively small sample size. All post-OLT PSC patients were on systemic immunosuppression, which included low-dose (≤10 mg prednisolone) with or without cyclosporine or tacrolimus. Additionally, OV was not used as a first-line therapy for induction or maintenance of remission of the colitis, and was used as an adjuvant therapy when conventional treatment options for management of IBD had failed.

Thus, the patient characteristics with the most favorable response, dosage, formulation, duration, and long-term impact of OV in patients with PSC with or without an associated IBD need to be determined by prospective, longer-term, randomized controlled trials. In addition, studies should aim to identify characteristics of gastrointestinal microbiota in PSC patients responding to antibiotic therapy and remain in remission after discontinuation of antibiotic therapy. Characterization of the interdependence between microbiota, immune function, liver function, and the antibiotic-induced clinical remission will allow to individualize therapy by defining patients who are likely to respond to antibiotic therapy (and who stay in remission). The findings also will enable the development of intervention strategies that specifically target microbes potentially involved in the pathophysiology of PSC.

## Data Availability

All data are incorporated into the article and any further data requested (underlying this article) will be shared on reasonable request to the corresponding author.

## Declarations

### Guarantor of the article

Prof Gerald Holtmann

### Disclaimer

The author is solely responsible for the data and the contents of the paper. In no way is the honorary editor in chief, editorial board members, the Indian Society of Gastroenterology, or the printer/publishers responsible for the results/findings and content of this article Indian Journal of Gastroenterology.
